# Screening of ferroptosis-related genes in sepsis-induced liver failure and analysis of immune correlation

**DOI:** 10.7717/peerj.13757

**Published:** 2022-07-29

**Authors:** Qingli Chen, Luxiang Liu, Shuangling Ni

**Affiliations:** 1 Department of Emergency Medicine, Lishui City People’s Hospital, Lishui, Zhejiang Province, China; 2 Department of Infectious Disease, Lishui City People’s Hospital, Lishui, Zhejiang Province, China

**Keywords:** Sepsis, Liver failure, Ferroptosis-related gene, Immunoassay, Animal experimental verification

## Abstract

**Purpose:**

Sepsis-induced liver failure is a kind of liver injury with a high mortality, and ferroptosis plays a key role in this disease. Our research aims to screen ferroptosis-related genes in sepsis-induced liver failure as targeted therapy for patients with liver failure.

**Methods:**

Using the limma software, we analyzed the differentially expressed genes (DEGs) in the GSE60088 dataset downloaded from the Gene Expression Omnibus (GEO) database. Clusterprofiler was applied for enrichment analysis of DEGs enrichment function. Then, the ferroptosis-related genes of the mice in the FerrDb database were crossed with DEGs. Sepsis mice model were prepared by cecal ligation and perforation (CLP). ALT and AST in the serum of mice were measured using detection kit. The pathological changes of the liver tissues in mice were observed by hematoxylin-eosin (H & E) staining. We detected the apoptosis of mice liver tissues using TUNEL. The expression of Hmox1, Epas1, Sirt1, Slc3a2, Jun, Plin2 and Zfp36 were detected by qRT-PCR.

**Results:**

DEGs analysis showed 136 up-regulated and 45 down-regulated DEGs. Meanwhile, we found that the up-regulated DEGs were enriched in pathways including the cytokine biosynthesis process while the down-regulated DEGs were enriched in pathways such as organic hydroxy compound metabolic process. In this study, seven genes (Hmox1, Epas1, Sirt1, Slc3a2, Jun, Plin2 and Zfp36) were obtained through the intersection of FerrDb database and DEGs. However, immune infiltration analysis revealed that ferroptosis-related genes may promote the development of liver failure through B cells and natural killer (NK) cells. Finally, it was confirmed by the construction of septic liver failure mice model that ferroptosis-related genes of Hmox1, Slc3a2, Jun and Zfp36 were significantly correlated with liver failure and were highly expressed.

**Conclusion:**

The identification of ferroptosis-related genes Hmox1, Slc3a2, Jun and Zfp36 in the present study contribute to our understanding of the molecular mechanism of sepsis-induced liver failure, and provide candidate targets for the diagnosis and treatment of the disease.

## Introduction

Around 30 million people are affected by sepsis worldwide every year (Huang, Cai & Su, 2019). Multiple factors contribute to the pathogenesis of sepsis, and the liver plays a central role in sepsis (Kasper et al., 2020). Revealed by some researches, many acute phase proteins and inflammatory cytokines are produced in sepsis-induced liver failure (Horvatits et al., 2019). Pathogens or toxins in sepsis reach the liver through blood, and then the liver macrophages can recognize and phagocytose pathogens (Kasper et al., 2020). However, the released pro-inflammatory cytokines will in turn induce liver inflammation, thereby activating apoptosis signaling pathway and leading to liver failure (Strnad et al., 2017). Although progresses have been made in the medical level, sepsis is still not effectively treated (Singer et al., 2016). Therefore, our research conducted an in-depth study on sepsis-induced liver failure.

Ferroptosis is a novel mechanism of cell death regulation (Stockwell et al., 2017). Ferroptosis was also revealed in liver injury, inflammation and other diseases (Wang et al., 2017). Irisin can inhibit ferroptosis and restore mitochondrial function in sepsis (Wei et al., 2020). Downregulating HO-1 expression and iron concentration can reduce ferroptosis, thereby attenuating myocardial cell injury in sepsis (Wang et al., 2020a). In addition, ferroptosis exert its action in regulating liver diseases, including liver failure, hepatocellular carcinoma, liver fibrosis, and liver ischemic injury (Kim, Cho & Ki, 2020). Therefore, we hope through bioinformatics technology to quickly find ferroptosis-related genes in sepsis-induced liver failure, making them possibly being used for the early diagnosis of sepsis-induced liver failure and thus providing new ideas for the clinical treatment of the disease.

In this study, we first analyzed the differentially expressed genes (DEGs) in the GSE60088 dataset using the limma software package. Then, we take intersection of the ferroptosis-related genes of mice in the FerrDb database with DEGs to obtain ferroptosis-related genes. Finally, we verified the expression of ferroptosis-related genes in liver tissues by constructing sepsis-induced liver failure mice model. In summary, the ferroptosis-related genes we studied in this research can provide new therapeutic targets for patients with sepsis-induced liver failure.

## Methods

### Data acquisition

We downloaded the original CEL data of GSE60088 from the GEO database (http://www.ncbi.nlm.nih.gov/geo/), which contained the gene data of 27 mice sepsis models (sepsis model was established by Staphylococcus aureus) and the controls. Five liver failure samples and three control samples were selected for subsequent analysis. The CEL file was first processed using the affy software package, and the RMA algorithm was applied for standardization.

### DEGs analysis

We performed differential gene expression analysis of the liver failure in the GSE60088 dataset using the limma (Version: 3.42.2) software package (Ritchie et al., 2015), and the Volcano plot and heatmap were correspondingly drawn. Adjusted *P* values (adj. P) < 0.05 and —logFC—>1 were set as the cutoff criterion to select DEGs for every dataset microarray, respectively (Chen et al., 2021).

### Functional enrichment analysis

Gene Ontology (GO) (Chen et al., 2017) and Kyoto Encyclopedia of Genes and Genomes (KEGG) (Wrzodek, Drager & Zell, 2011) pathway enrichment analysis was conducted using ClusterProfiler for both the up-regulated and down-regulated genes in the GSE60088 dataset (Xu et al., 2021), and the enrichment results were screened with padj < 0.05 as the threshold.

### Identification of ferroptosis-related genes

We obtained a total of 107 ferroptosis-related genes of mice from the FerrDb (http://www.zhounan.org/ferrdb/legacy/index.html) database. The ferroptosis-related genes related to liver failure were obtained by crossing the ferroptosis-related genes with the DEGs.

### Validation of ferroptosis-related genes

We downloaded the GSE199598 dataset (containing four control samples and four sepsis liver tissue samples from mice) and the GSE95233 dataset (containing 22 control samples and 102 samples from patients with sepsis) from the GEO. The R package ‘ggplot2’ was used to draw the expression boxplots of ferroptosis-related genes for validation (Wu et al., 2021).

### Immune infiltration analysis

Infiltration analysis of 22 types of immune cells was performed on the liver failure group and the control group using “CIBERSORT” (https://cibersort.stanford.edu/) in the R package (*P* < 0.05) (Newman et al., 2015). Differential immune cells in the liver failure group and the control group were screened by boxplot. Meanwhile, correlation analysis of 22 types of immune cells was conducted using R software.

### Preparation of sepsis-induced liver failure mice model

The mice model of CLP-induced sepsis-induced liver failure was established using the C57BL/6 male mice aged from six to eight weeks (Hangzhou Medical College, China, Hangzhou). The mice were housed in ventilated cages (22 °C, 40–60% humidity, 12-hour light/dark cycle) with food and water. The animal experiment in this research has been approved by the Institutional Animal Care and Use Committee (IACUC), Zhejiang Provincial Committee for Laboratory Animal (ZJCLA) (Approval No. ZJCLA-IACUC-20010070). First, the cecum of mice was ligated after anesthesia and punctured with a needle. Then, we extruded the feces of mice from the puncture wound and sutured the wound. After the operation, sterile saline solution was subcutaneously injected in mice (0.9%). The successful modeling of sepsis-induced liver failure mice model was indicated if the systemic inflammatory response reached the peak at 12 h after the operation, and the mice had symptoms such as erect hair, diarrhea and pyuria. After 12 h of CLP-induced sepsis-induced liver failure in the mice model, the whole body serum of the mice was collected by cardiac puncture for subsequent experiments. All mice were euthanized by CO_2_ asphyxiation after the experiment. In addition, mice without ligation and puncture were included in the sham operation group (Sham group), namely the control group.

### Serum ALT and AST measurement

Serum ALT and AST were determined using the detection kit (Invitrogen, Waltham, MA, USA), and the contents of ALT and AST in plasma were determined according to the manufacturer’s instructions.

### Hematoxylin-eosin (H & E) staining

The liver tissues of mice were fixed in 4% paraformaldehyde for 24 h, embedded in paraffin and prepared into 5 µm slices. Then, the liver tissue slices were stained with hematoxylin and eosin (Invitrogen, Waltham, MA, USA) reagent. Finally, the pathological changes of mice liver tissues were observed under a microscope.

### TUNEL detection

The apoptosis of mice liver tissues was detected using TUNEL (Invitrogen, Waltham, MA, USA). According to the operating instruction manual of TUNEL detection kit (Merck Millipore, Darmstadt, Germany), the liver tissue sections of mice were stained with TUNEL reagent to determine the apoptosis of liver tissues. Finally, the apoptosis was observed under an optical microscope.

### qRT-PCR assay

The total RNA of the samples was obtained using the Trizol kit (Invitrogen, Waltham, MA, USA), and cDNA was synthesized using the reverse transcription kit (Invitrogen, Waltham, MA, USA) and detected by real-time quantitative PCR. With GAPDH as the internal reference, the relative gene expression levels of Hmox1, Epas1, Sirt1, Slc3a2, Jun, Plin2 and Zfp36 were calculated by 2^−ΔΔ*CT*^ method. The primer pairs applied for qRT-PCR in Table 1.

### Statistical processing

GraphPad prism 9.0 software (Graphpad, San Diego, CA, USA) was applied for *t* test or one-way ANOVA (analysis of variance), and *P* < 0.05 was considered statistically significant.

## Results

### DEGs analysis

**Figure 1:**
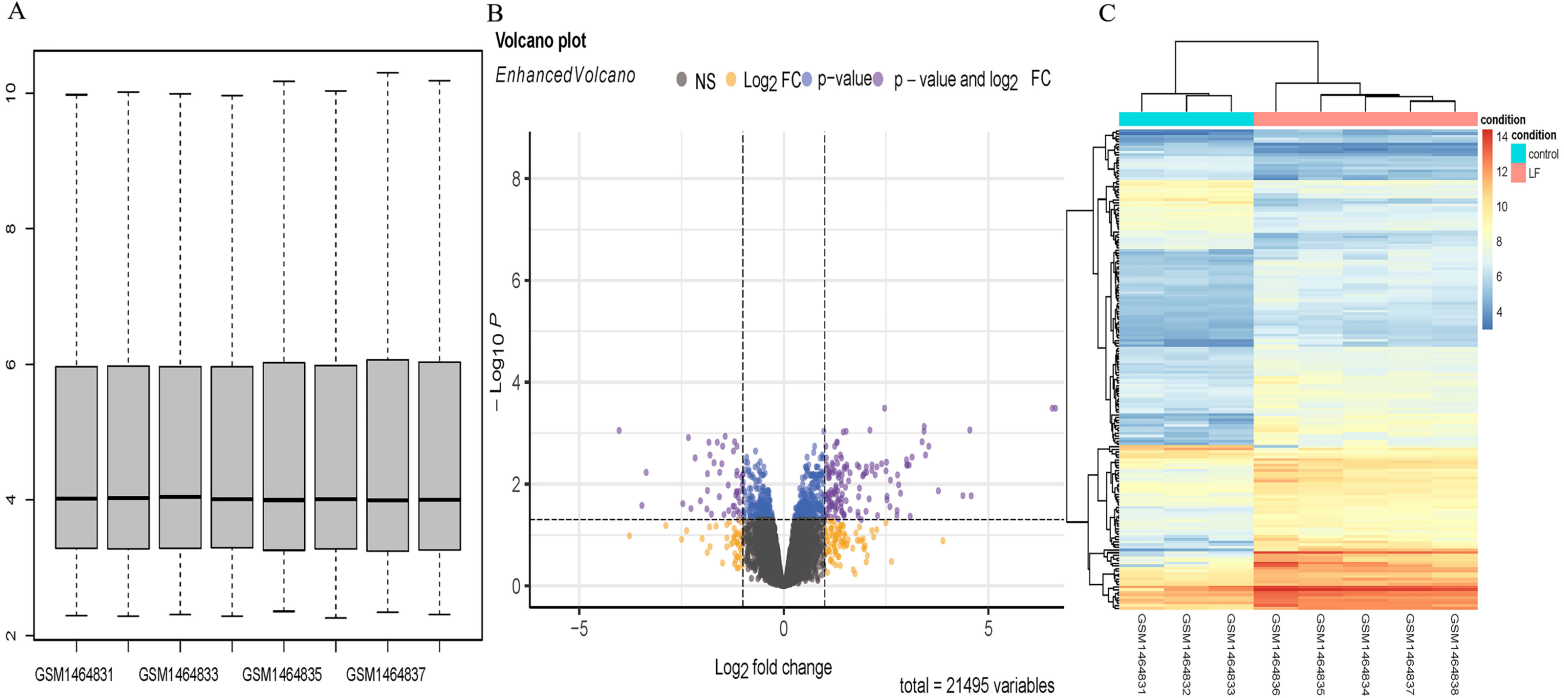
DEGs analysis. (A) Boxplot diagram of the DEGs in the GSE60088 dataset. (B) Volcano plot of the DEGs in the GSE60088 dataset. (C) Heatmap of the DEGs in the GSE60088 dataset.

Five sepsis models and three controls of mice in the GSE60088 dataset were processed by the affy software package to obtain the boxplot (Fig. 1A). A total of 181 DEGs (Supplementary Material 1) in the sepsis models and the controls were revealed by analysis of the Volcano plot and Heatmap of DEGs, including 136 up-regulated and 45 down-regulated DEGs (Figs. 1B, 1C).

**Table 1:** The primer pairs applied for qRT-PCR.

	Forward primer 5′–3′	Reverse primer 5′–3′
Hmox1	ACCGCCTTCCTGCTCAACATTG	CTCTGACGAAGTGACGCCATCTG
Epas1	ACAACCTCCTCCCACTCCTTTCC	TCCGAGAGTCCCGCTCAATCAG
Sirt1	CCAGACCTCCCAGACCCTCAAG	GTGACACAGAGACGGCTGGAAC
Slc3a2	GCAGGACGGTGTGGATGGTTTC	ATTCTGCCACTCAGCCAAGTACAAG
Jun	CTTCTACGACGATGCCCTCAACG	GCCAGGTTCAAGGTCATGCTCTG
Plin2	GCAACAGAGCGTGGTGATGAGAG	CTGACATAAGCGGAGGACACAAGG
Zfp36	TCTGAGTGACAAGTGCCTACCTACC	GTCCCCACAGCAATGAGCAGTC
GAPDH	AGGAGAGTGTTTCCTCGTCC	TGCCTGAGTGGAGTCATAC

### Functional enrichment analysis

**Figure 2:**
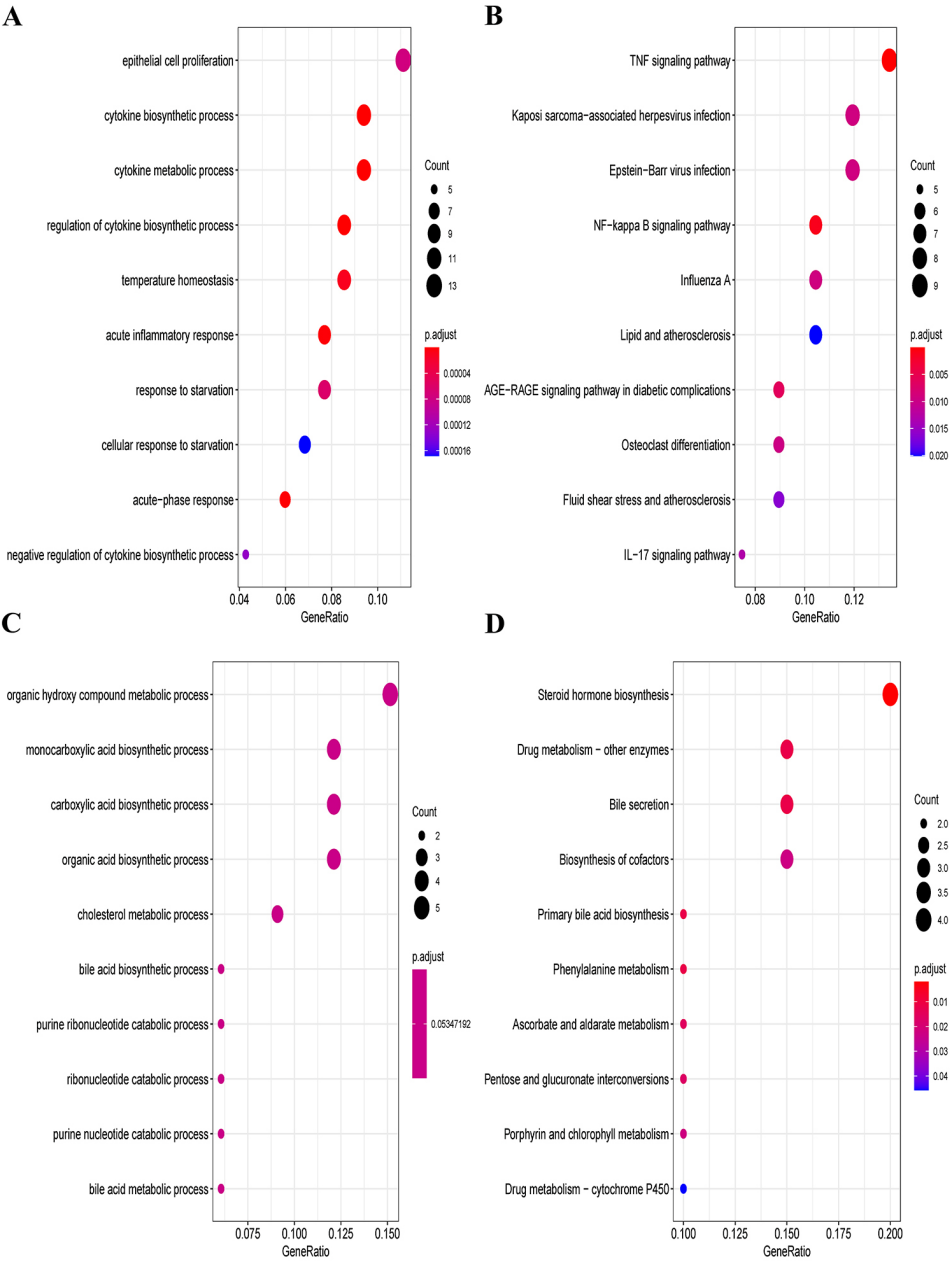
GO and KEGG enrichment analysis of DEGs. (A) GO enrichment analysis of the up-regulated DEGs. (B) KEGG enrichment analysis of the up-regulated DEGs. (C) GO enrichment analysis of the down-regulated DEGs. (D) KEGG enrichment analysis of the down-regulated DEGs.

Furthermore, we explored the functions and enrichment pathways of DEGs in this research, and conducted GO and KEGG enrichment analysis on the up- and down-regulated DEGs in the GSE60088 dataset. Great differences in the functions of DEGs were indicated by the results of analysis. The up-regulated DEGs were mainly enriched in pathways such as cytokine production process, acute inflammatory response, TNF signaling pathway, NF-kappa B signaling pathway and IL-17 signaling pathway (Figs. 2A, 2B, Supplementary Material 2 and Supplementary Material 3). The down-regulated DEGs were mainly enriched in pathways such as organic hydroxy compound metabolic process, monocarboxylic acid production process, steroid hormone production, drug metabolism-other enzymes and bile secretion (Figs. 2C, 2D, Supplementary Material 4 and Supplementary Material 5).

### Screening of ferroptosis-related genes

**Figure 3:**
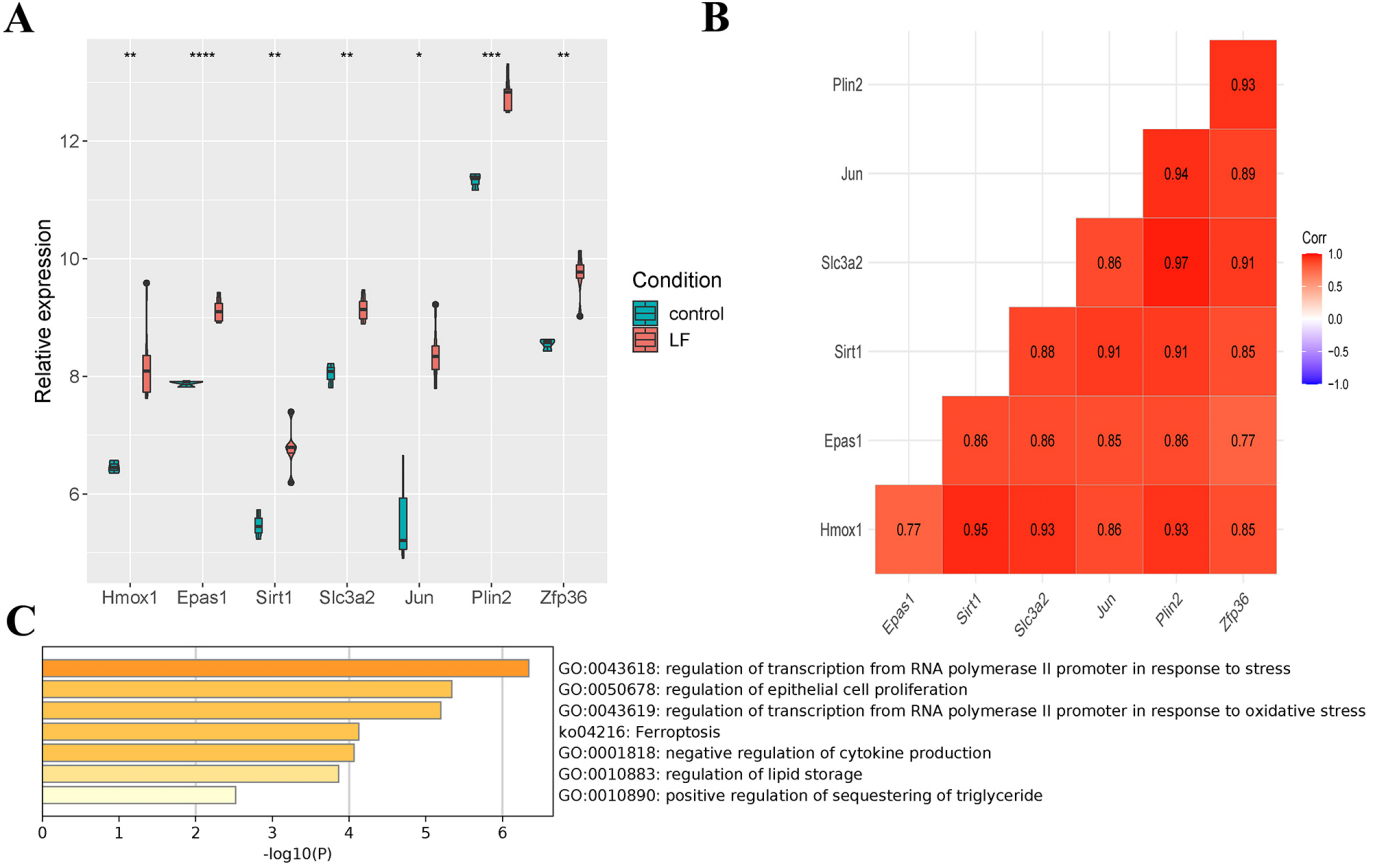
Screening of ferroptosis-related genes. (A) Expression of ferroptosis-related genes. (B) Correlation analysis of ferroptosis-related genes. (C) Enrichment analysis of the seven genes.

Seven differentially expressed ferroptosis-related genes (Hmox1, Epas1, Sirt1, Slc3a2, Jun, Plin2 and Zfp36) were obtained through the intersection of ferroptosis-related genes in the FerrDb database with DEGs in the GSE60088 dataset, and all of these genes were up-regulated in liver failure (Fig. 3A). At the same time, a significant positive correlation between the seven genes was indicated by Pearson correlation analysis, and the correlation between Slc3a2 and Plin2 was the strongest (Cor = 0.97, Fig. 3B). Then, we performed enrichment analysis on the seven genes using the metascape software (Fig. 3C, Table 2). Results showed that Hmox1, Sirt1 and Slc3a2 were enriched in the ko04216 ferroptosis pathway; Hmox1, Epas1, Sirt1 and Jun were enriched in the regulation of transcription from RNA polymerase II promoter in response to stress; Hmox1, Epas1, Sirt1, Slc3a2, Jun and Zfp36 were enriched in the regulation of epithelial cell proliferation; Hmox1, Sirt1 and Zfp36 were enriched in the negative regulation of cytokine production.

**Table 2:** Enrichment pathways of the seven genes.

GO Terms	Gene
GO:0043618 regulation of transcription from RNA polymerase II promoter in response to stress	Hmox1, Epas1, Sirt1, Jun
GO:0050678 regulation of epithelial cell proliferation	Hmox1, Epas1, Sirt1, Slc3a2, Jun, Zfp36
GO:0043619 regulation of transcription from RNA polymerase II promoter in response to oxidative stress	Hmox1, Epas1, Jun, Zfp36
ko04216 Ferroptosis	Hmox1, Sirt1, Slc3a2
GO:0001818 negative regulation of cytokine production	Hmox1, Sirt1, Zfp36
GO:0010883 regulation of lipid storage	Sirt1, Plin2
GO:0010890 positive regulation of sequestering of triglyceride	Slc3a2, Plin2

### Validation of ferroptosis-related genes

**Figure 4:**
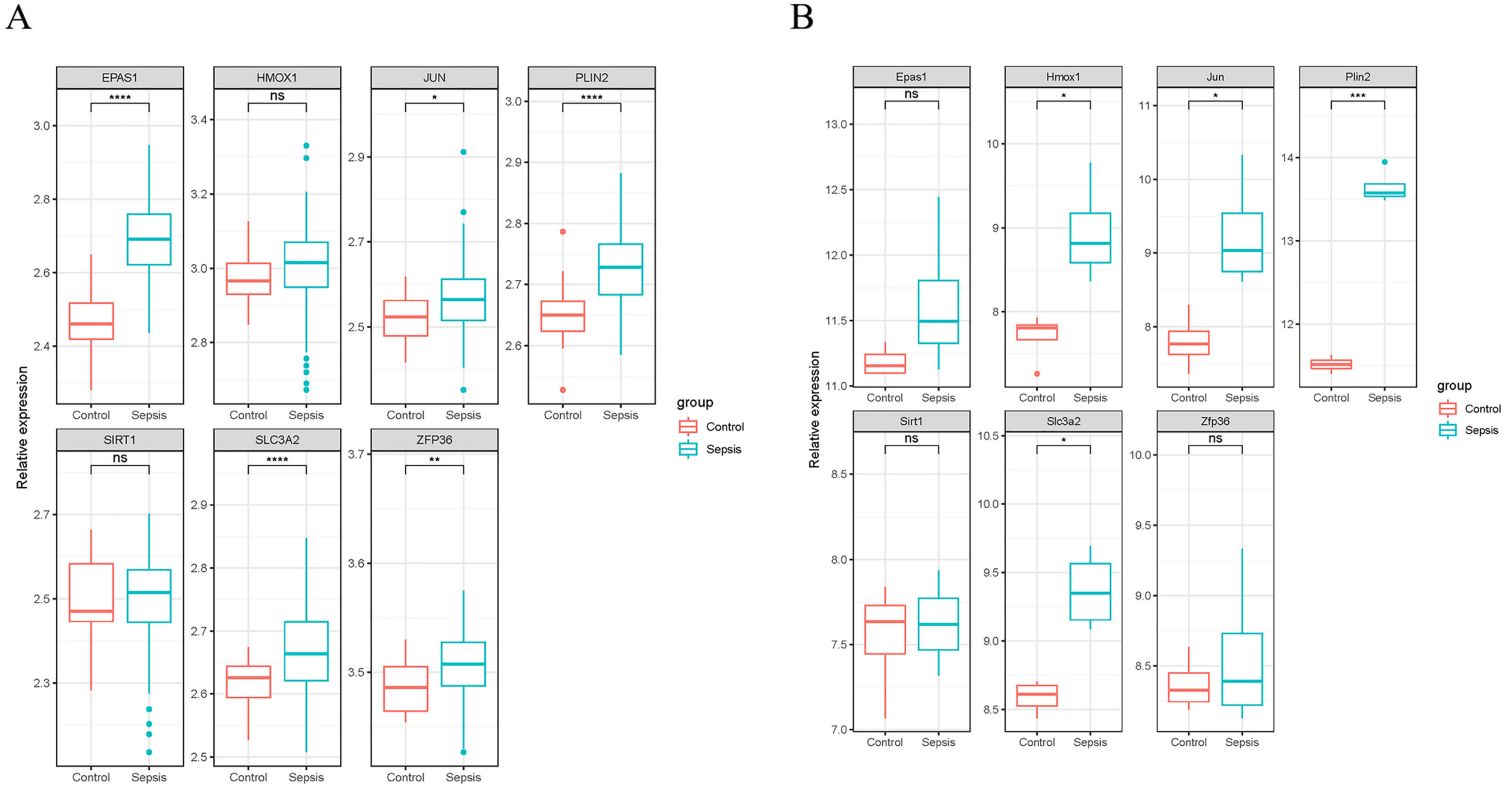
Expression of seven ferroptosis-related genes. (A) Expression of seven ferroptosis-related genes in the GSE199598 dataset. (B) Expression of seven ferroptosis-related genes in the GSE95233 dataset.

We validated the expression of the seven ferroptosis-related genes in GSE199598 (containing four control samples and four sepsis liver tissue samples from mice) and GSE95233 (containing 22 control samples and 102 samples from patients with sepsis) datasets. According to the validation results of the GSE199598 dataset, the expression levels of the seven ferroptosis-related genes (Hmox1, Epas1, Sirt1, Slc3a2, Jun, Plin2 and Zfp36) were all up-regulated, which was consistent with the results of the GSE60088 dataset (Fig. 4A). In addition, based on the validation results of the GSE95233 dataset, the expression levels of the seven ferroptosis-related genes (Hmox1, Epas1, Sirt1, Slc3a2, Jun, Plin2 and Zfp36) were also significantly up-regulated, and Sirt1 and Zfp36 were not significant (Fig. 4B), which was still consistent with the results from the GSE60088 dataset.

### Immune infiltration analysis

**Figure 5:**
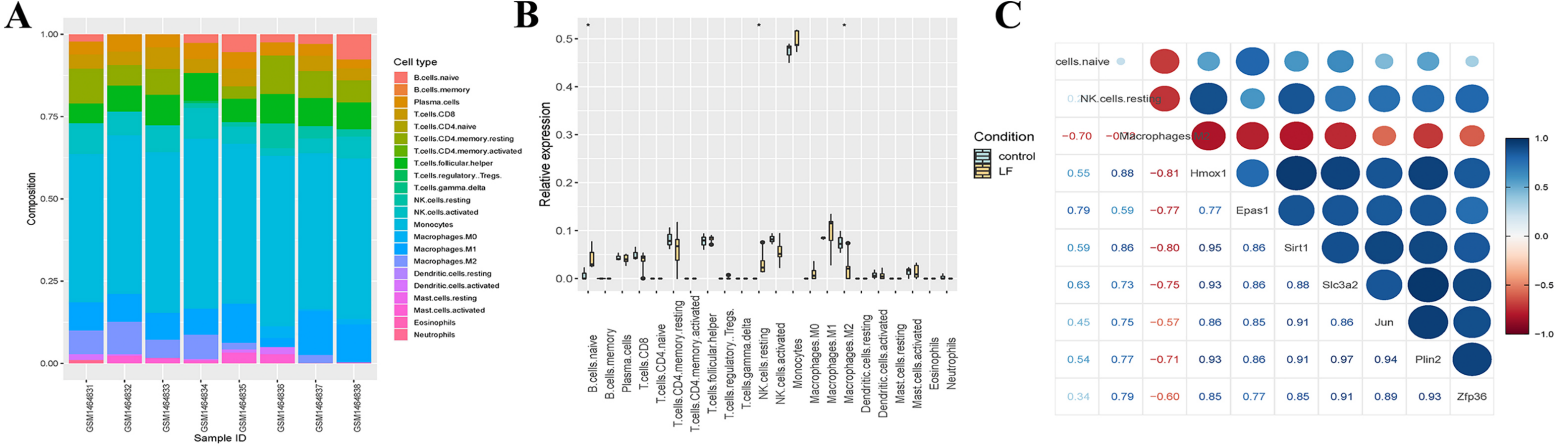
Analysis of the immune infiltration. (A) Immune infiltration strip map. (B) Differential immune cell boxplot map between the liver failure group and the control group. (C) Correlation analysis between immune cells and ferroptosis-related genes.

We also analyzed the immune infiltration of the samples, and the immune infiltration bar chart and the boxplot of the differential immune cells between the liver failure group and the control group were drawn accordingly (Figs. 5A, 5B). Significant differences in B cells, natural killer (NK) cells and M2 macrophages were revealed by further analysis, and the number of B cells and NK cells in the liver failure group increased significantly, while the number of M2 macrophages decreased. Besides, we performed correlation analysis on the three kinds of differential immune cells and seven differentially expressed ferroptosis-related genes (Fig. 5C). These results revealed that B cells and NK cells were positively correlated with the seven ferroptosis-related genes, and the correlation between NK cells and Hmox1 was the strongest (cor = 0.88). However, M2 macrophages was negatively correlated with the ferroptosis-related genes, and the negative correlation between M2 macrophages and Hmox1 was the strongest (cor = −0.81).

### Expression of ferroptosis-related genes verified by animal experiments

**Figure 6:**
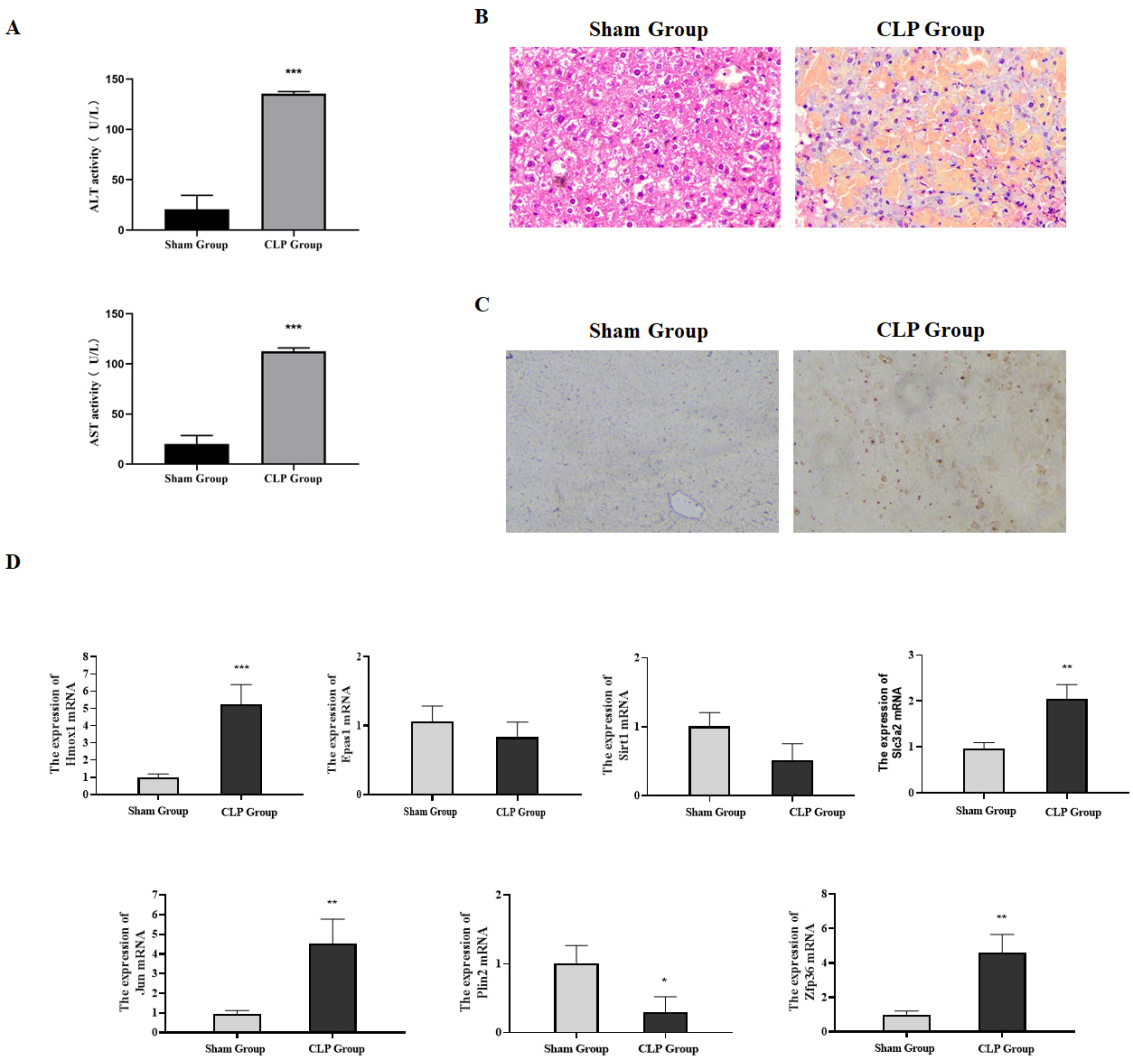
Expression of ferroptosis-related genes verified through animal experiments. (A) Determination of serum ALT and AST in mice, compared with the Sham group, ^***^P < 0.001. (B) H & E detection of the pathological changes of mice liver tissues. (C) The apoptosis in the liver tissues of mice detected by TUNEL. (D) The expression of ferroptosis-related genes in the liver tissues detected by qRT-PCR, compared with the Sham group, ^*^P < 0.05, ^**^P < 0.01, ^***^P < 0.001.

Our research verified the expression of ferroptosis-related genes by constructing a sepsis-induced liver failure mice model. Firstly, serum biochemical markers ALT and AST reflecting the liver function were analyzed. Compared with the sham group, the levels of serum ALT and AST in the CLP group were significantly increased (Fig. 6A, *P* < 0.05). Meanwhile, the pathological changes of the liver tissues in mice were observed by H & E staining. Focal and extensive necrosis was found in the liver tissues of mice in the CLP group (Fig. 6B). Besides, through TUNEL staining, the apoptosis rate in the liver tissues of mice in the CLP group was found to be higher than that in the Sham group (Fig. 6C). The mice model was successfully established in this study.

The qRT-PCR results showed that compared with the sham group, the mRNA expressions of Hmox1, Slc3a2, Jun and Zfp36 in the CLP group were significantly up-regulated, which was consistent with the conclusion drawn from bioinformatics, while the expressions of Sirt1, Epas1 and Plin2 were down-regulated (Fig. 6D). In summary, our research confirmed through animal experiments that ferroptosis-related genes Hmox1, Slc3a2, Jun and Zfp36 were significantly correlated with and highly expressed in liver failure.

## Discussion

Sepsis is a kind of host system reaction caused by pathogenic microorganisms in blood circulation, causing severe systemic inflammation (Hawiger, Veach & Zienkiewicz, 2015). Macrophages are activated under the stimulation of cytokines in sepsis such as macrophage colony-stimulating factors, TNF-α, pathogenic microorganisms and chemical mediators, and then the activated macrophages phagocytize and kill a variety of pathogens and present antigens (Xing et al., 2012). Now, the incidence of sepsis is still increasing (Kumar, 2018). Therefore, our research hope to obtain new therapeutic targets for sepsis-induced liver failure.

Ferroptosis plays a key role in sepsis organ damage (Li et al., 2020). Therefore, we screened the ferroptosis-related genes (Hmox1, Epas1, Sirt1, Slc3a2, Jun, Plin2 and Zfp36) in this study through bioinformatics analysis, and found that these genes were highly expressed in sepsis-induced liver failure model. At the same time, we verified in the GSE199598 dataset (mouse) and the GSE95233 dataset (human) that the expression levels of Hmox1, Epas1, Sirt1, Slc3a2, Jun, Plin2 and Zfp36 were all up-regulated, which were in line with the results of the GSE60088 dataset. In addition, it was found through immune infiltration analysis in this study that ferroptosis-related genes may promote the development of sepsis-induced liver failure through B cells and NK cells.

B cells are considered to have protective functions in sepsis, including antibody-dependent and non-dependent mechanisms (Kelly-Scumpia et al., 2011). However, B cells of sepsis undergo massive apoptosis, and release PAMP and DAMP (Hotchkiss et al., 2002; Jensen et al., 2021). Some researches found that serum IgM and IgG antibody concentrations are elevated under sepsis induction (Nicolai et al., 2020b). Reactive antibodies of B cells dominate in sepsis among the main humoral immune responses in the human body (Nicolai et al., 2020a).

NK cells are a kind of important natural immune cells (Otto et al., 2011). Severe infections and even deaths can be caused by the increase of T and NK cell apoptosis in sepsis (Athie-Morales, O’Connor & Gardiner, 2008). In addition, IL-18R of NK cells is reduced in sepsis mice (Hiraki et al., 2012). However, in another study, mice with NK cells deficiency demonstrate lower survival rate and higher levels of pro-inflammatory cytokines (Guo et al., 2017).

Besides, neutrophils and natural killer T cells are also involved in the sepsis-related liver failure (Strnad et al., 2017; Wang, Yin & Yao, 2014). These cells exacerbate liver injury in sepsis by secreting pro-inflammatory cytokines (Heymann & Tacke, 2016). Immune infiltration analysis showed that Hmox1 was closely associated with NK cells in Alzheimer’s disease (Wu et al., 2021). Upregulation of Slc3a2 enhances the tumor specificity of NK cells (Nachef et al., 2021). Deletion of Slc3a2 in B cells disrupts processes such as B cell proliferation, plasma cell formation, and antibody secretion (Cantor et al., 2011). The Zfp36 gene is expressed during B-cell development and promotes the recruitment of Zfp36 gene target mRNAs (Akiyama & Yamamoto, 2021). Meanwhile, bioinformatics analysis indicated that the expression levels of Jun and Zfp36 were correlated with NK-cell infiltration (Dong et al., 2022). In general, the conclusions drawn from these reports are consistent with the results in our research, that is, Hmox1, Epas1, Sirt1, Slc3a2, Jun, Plin2 and Zfp36 collectively promote the development of sepsis-induced liver failure by interacting with B cells and NK cells.

We also used a mouse model of sepsis-induced liver failure, and confirmed through qRT-PCR detection that ferroptosis-related genes Hmox1, Slc3a2, Jun and Zfp36 were significantly correlated with sepsis-induced liver failure. Hmox1 is a kind of stress protein-encoded gene. In addition, some researches show that Hmox1 protects against heme injury (Ning et al., 2020), and can regulate Hmox1 protein expression in response to oxidative damage, including the oxidative damage in sepsis (Piantadosi et al., 2011; Vazquez-Armenta et al., 2013). Hmox1 was confirmed in this study playing an important role in sepsis.

Slc3a2 plays important roles in tumor growth and oxidative stress control (Digomann, Linge & Dubrovska, 2019). Upregulating Slc3a2 can activate the pro-inflammatory cytokines in lymphocyte effector and regulate cell metabolism, growth and proliferation (Nachef et al., 2021). T cell expansion can be prevented by the loss of Slc3a2 of effector molecules in NK cells (Cibrian et al., 2020). These reports are consistent with the results of immune infiltration analysis in this research.

Jun is a variety of gene encoding c-Jun protein and a key downstream target of JNK pathway (Vogt, 2002). However, c-jun has been considered as a key mediator in tumor progression (Ge et al., 2020; Miao & Ding, 2003; Wang et al., 2020b), playing antiapoptotic role in many tumors (Eferl et al., 2003; Vleugel et al., 2006). However, Jun has not been studied in sepsis, so we demonstrated Jun may be a new therapeutic target for sepsis-induced liver failure in this research.

Zfp36, as a kind of RNA-binding protein gene, is also one of the cytoplasmic mRNA regulators (Tiedje et al., 2016). Zfp36 expression is down-regulated in a variety of tumors (Montorsi et al., 2016). Interestingly, Zfp36 exerts influence in the treatment of hepatocellular carcinoma by regulating PRC1 (Chen et al., 2020), while it also plays an anti-tumor role through the knockdown of Zfp36 expression to affect inflammation, metabolism and cell proliferation (Krohler et al., 2019). Zfp36 protein binds to AU-rich elements in the 3′UTR of the corresponding mRNAs to inhibit the expression of inflammatory cytokines and promote the resolution of inflammation (Joe et al., 2020). Meanwhile, up-regulating the expression of Zfp36 gene can suppress the inflammatory response and induce the autophagy to clear bacteria in sepsis model mice, thereby improving sepsis outcomes (Joe et al., 2020). Our research also proved that Zfp36 is closely related to sepsis-induced liver failure. In summary, shown by the above reports, Hmox1, Slc3a2, Jun and Zfp36 play important roles in sepsis, which are consistent with our findings.

To sum up, the main advantage of our research lies in the screening of ferroptosis-related genes (including Hmox1, Slc3a2, Jun and Zfp36) in sepsis-induced liver failure. First, ferroptosis-related genes were screened through bioinformatics analysis in sepsis-induced liver failure. Second, we performed immune infiltration analysis on these ferroptosis-related genes and found that they may promote the development of liver failure through B cells and NK cells. In addition, we constructed a mouse model, and confirmed through H & E staining, TUNEL staining and qRT-PCR that Hmox1, Slc3a2, Jun and Zfp36 were significantly related to sepsis-induced liver failure. As a result, Hmox1, Slc3a2, Jun and Zfp36 may become new therapeutic targets for sepsis-induced liver failure in the future.

##  Supplemental Information

10.7717/peerj.13757/supp-1 Supplemental Information 1 Author Checklist

10.7717/peerj.13757/supp-2 Supplemental Information 2 Differential expression genes (DEGs)

10.7717/peerj.13757/supp-3 Supplemental Information 3 GO enrichment analysis of the up-regulated DEGs

10.7717/peerj.13757/supp-4 Supplemental Information 4 KEGG enrichment analysis of the up-regulated DEGs

10.7717/peerj.13757/supp-5 Supplemental Information 5 GO enrichment analysis of the down-regulated DEGs

10.7717/peerj.13757/supp-6 Supplemental Information 6 KEGG enrichment analysis of the down-regulated DEGs

10.7717/peerj.13757/supp-7 Supplemental Information 7 Raw data - ALT&AST

10.7717/peerj.13757/supp-8 Supplemental Information 8 Raw Data - RT-qPCR(ferroptosis)

10.7717/peerj.13757/supp-9 Supplemental Information 9 Raw data for Fig. 6 (HE-CLP)

10.7717/peerj.13757/supp-10 Supplemental Information 10 Raw data for Fig. 6 (HE-Sham)

10.7717/peerj.13757/supp-11 Supplemental Information 11 Raw data for Fig. 6 (Tunel)

## Additional Information and Declarations

### Data Availability

The following information was supplied regarding data availability: The datasets used and/or analyzed during the current study are available at GEO: GSE60088 and the FerrDb database (http://www.zhounan.org/ferrdb/legacy/operations/download.html).
