# Supplementary figures and images for: Screening of ferroptosis-related genes in sepsis-induced liver failure and analysis of immune correlation
Source: PeerJ. 2022 Jul 29;10:e13757. doi: 10.7717/peerj.13757 (PMC9341447; doi:10.7717/peerj.13757)

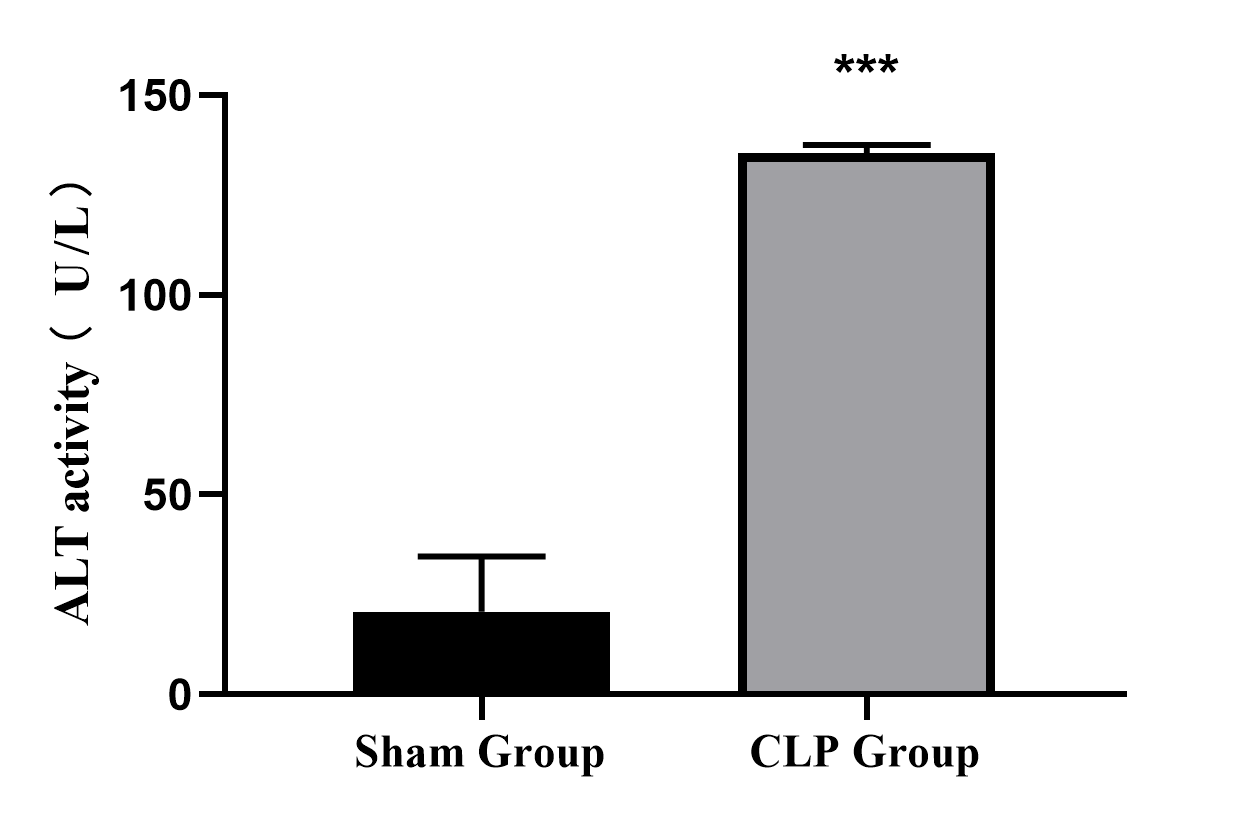

Supplement: Supplemental Information 7 [file peerj-10-13757-s007.zip › ALT&AST/ALT.tif]

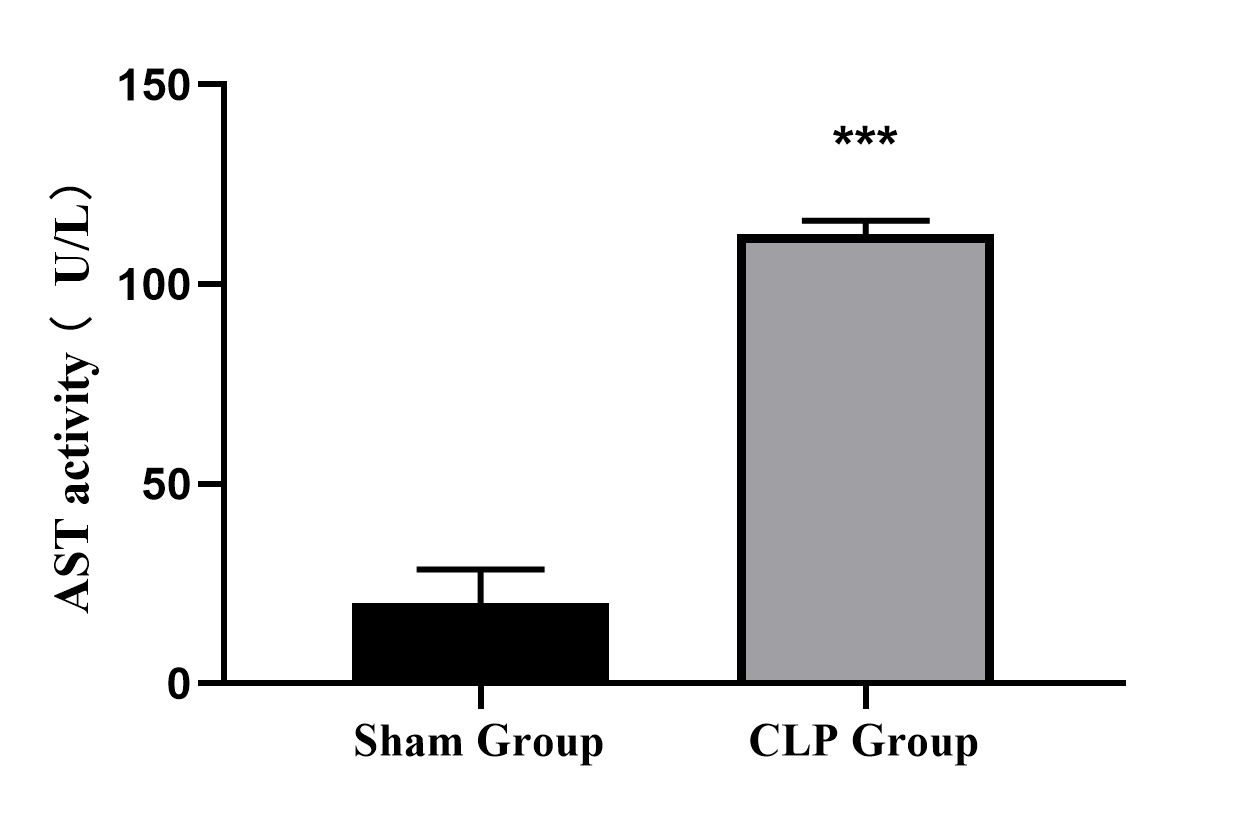

Supplement: Supplemental Information 7 [file peerj-10-13757-s007.zip › ALT&AST/AST.tif]

## Slide 1
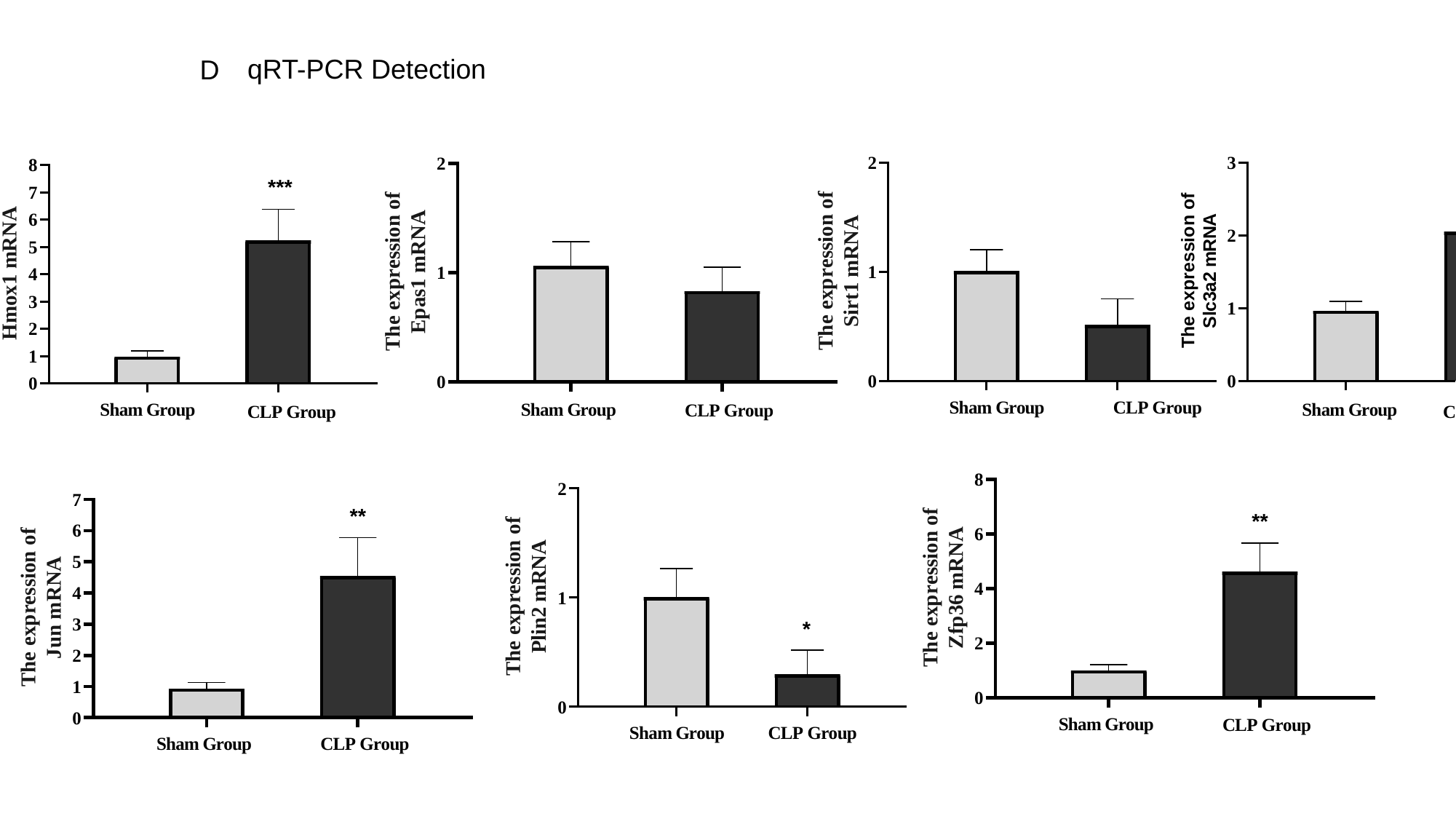

qRT-PCR Detection
D

Supplement: Supplemental Information 8 [file peerj-10-13757-s008.zip › RT-qPCR(ferroptosis)/qRT-PCR (bar chart).pptx]

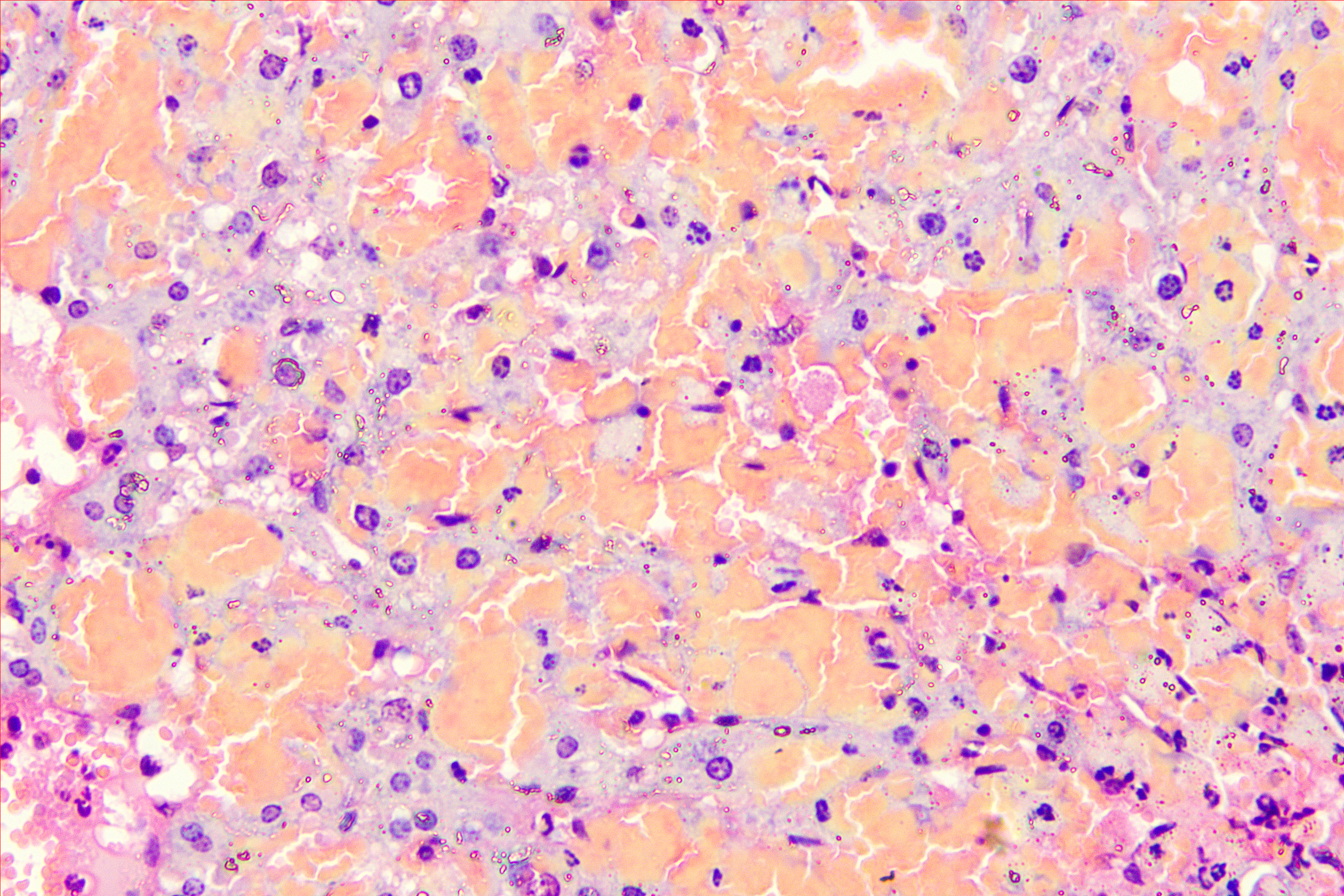

Supplement: Supplemental Information 9 [file peerj-10-13757-s009.tif]

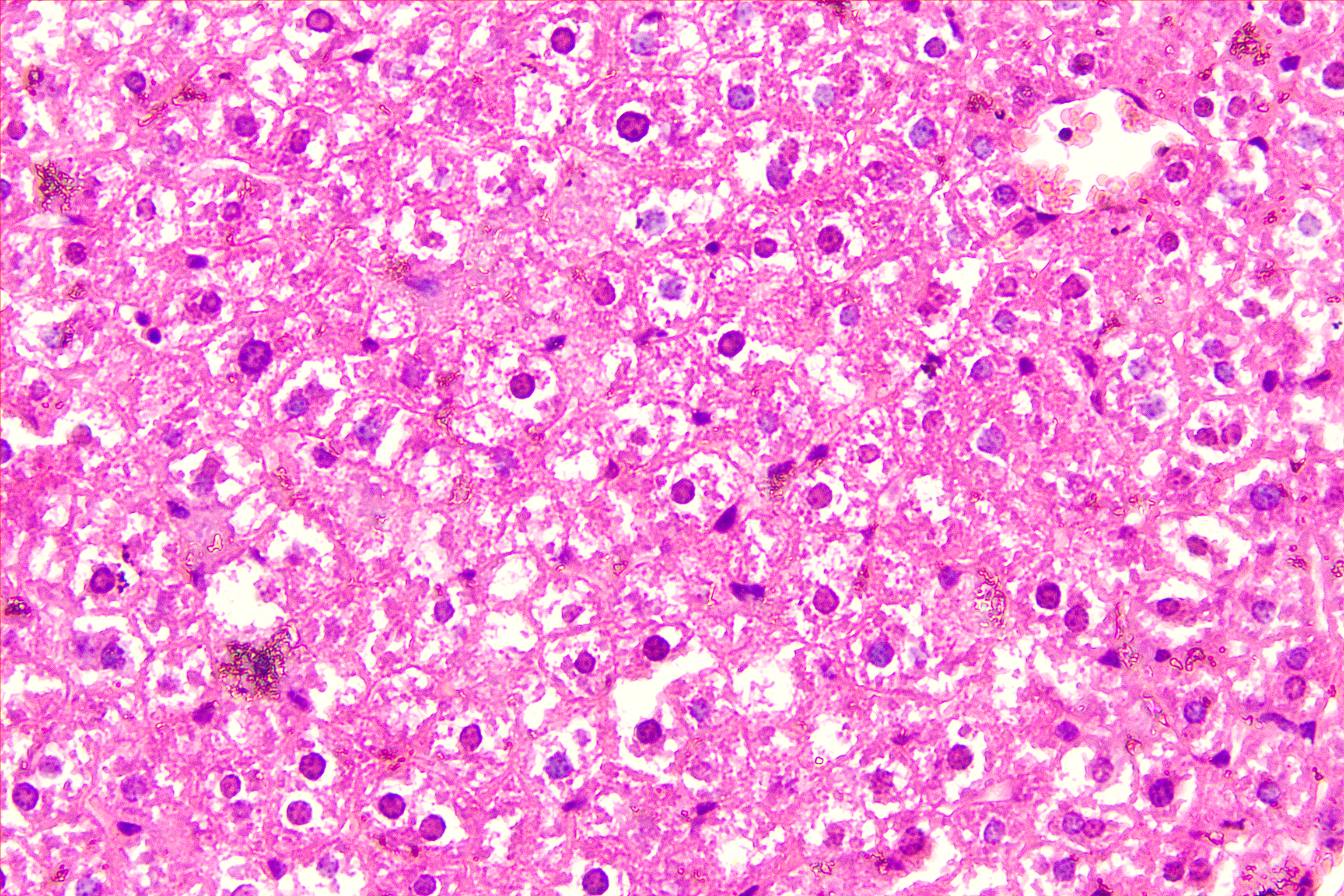

Supplement: Supplemental Information 10 [file peerj-10-13757-s010.tif]

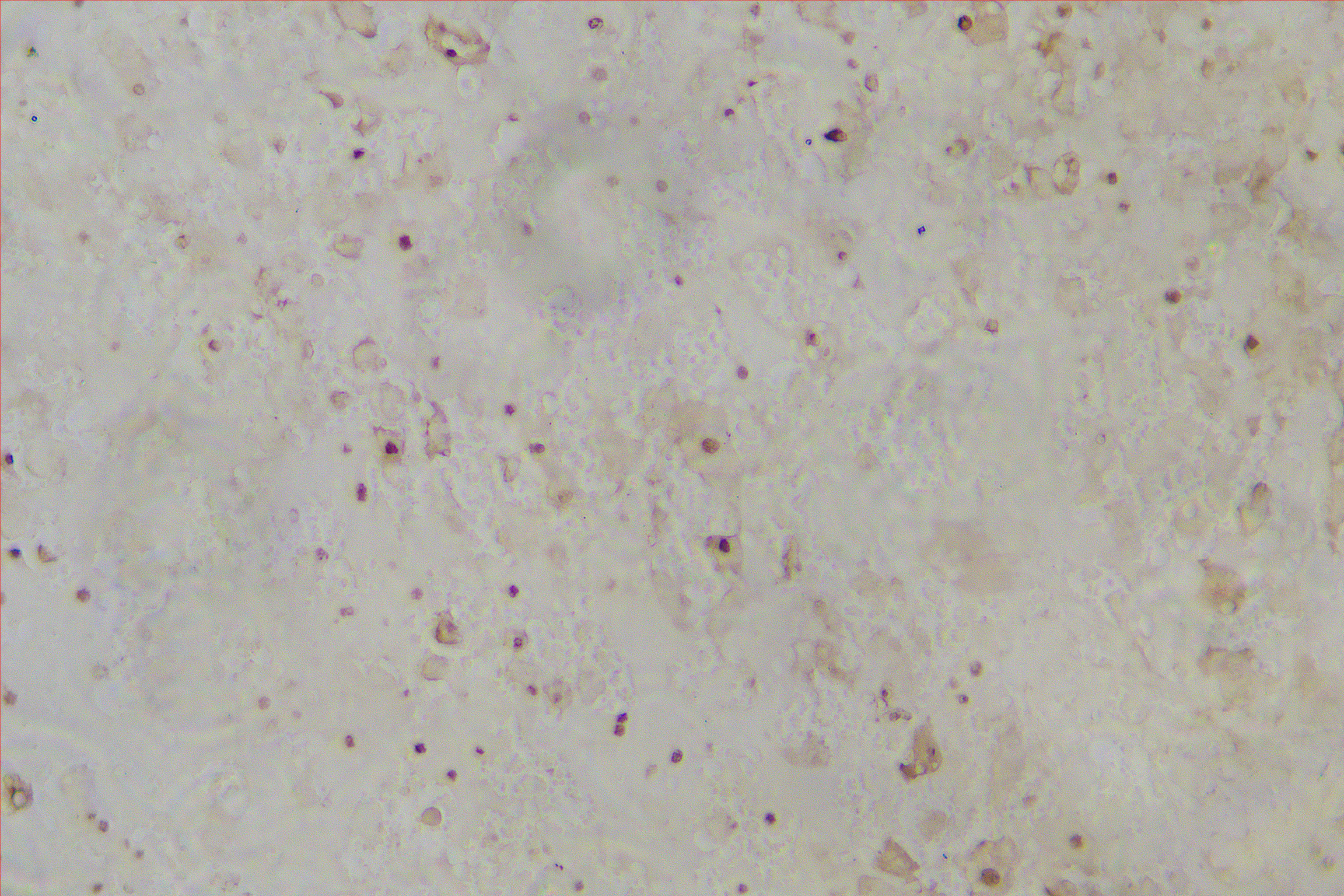

Supplement: Supplemental Information 11 [file peerj-10-13757-s011.zip › Tunel/CLP.tif]

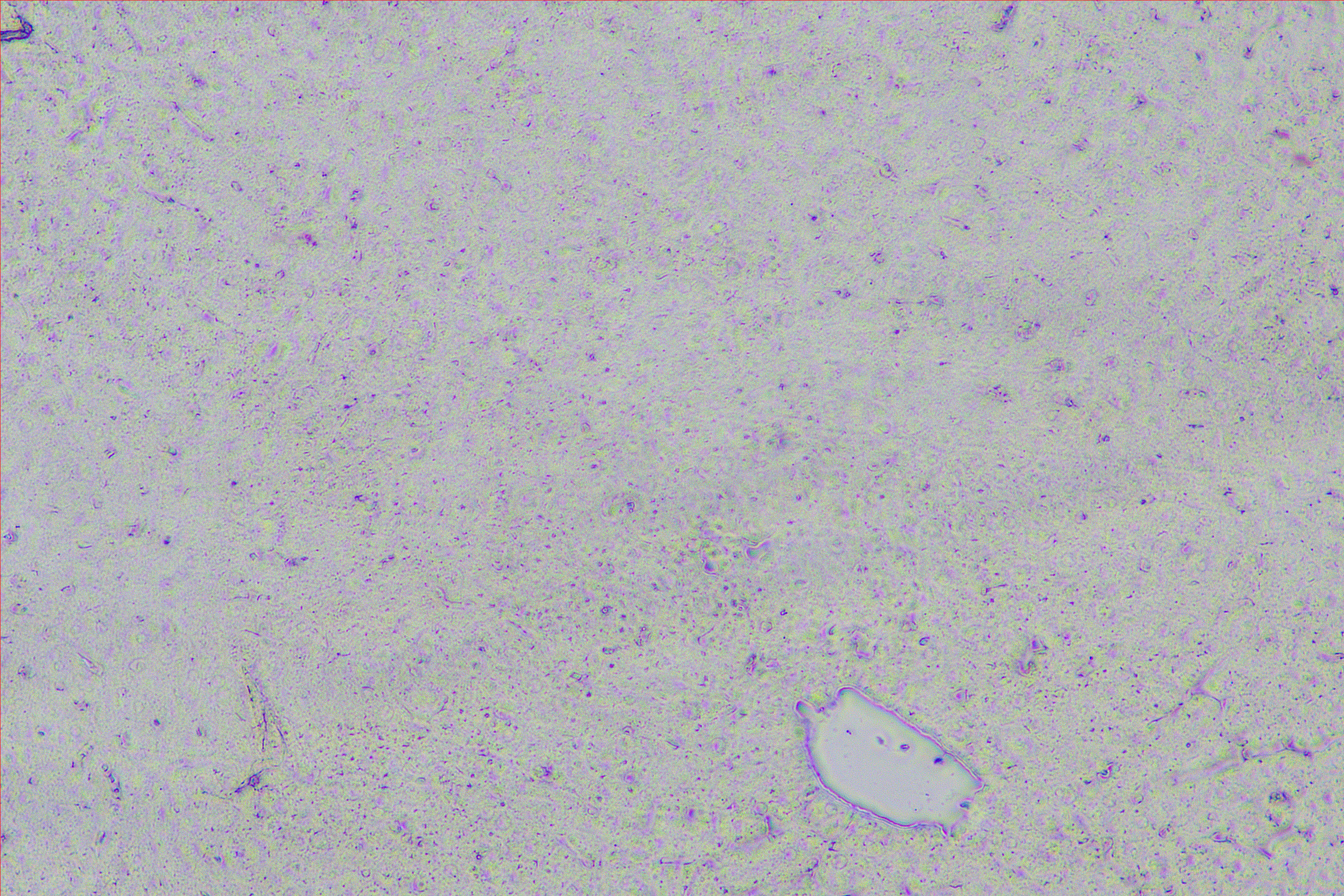

Supplement: Supplemental Information 11 [file peerj-10-13757-s011.zip › Tunel/Sham.tif]
